# Revision of failed traditional fundoplication using EsophyX^®^ transoral fundoplication

**DOI:** 10.1007/s00464-012-2542-7

**Published:** 2012-10-10

**Authors:** Reginald C. W. Bell, Rachel J. Hufford, Jacqueline Fearon, Katherine D. Freeman

**Affiliations:** Swedish Medical Center and SurgOne P.C., 401 W Hampden Place, Suite 230, Englewood, CO 80110 USA

**Keywords:** Fundoplication, EsophyX^®^, Gastroesophageal reflux, Laparoscopy, Nissen fundoplication, TIF

## Abstract

**Background:**

Laparoscopic revision of failed traditional fundoplication is difficult and involves risk of gastric, esophageal, and vagal nerve injury that is higher than that of the primary fundoplication. This study assessed feasibility and clinical outcomes of the transoral approach to revision of loose Nissen.

**Methods:**

Between November 2009 and August 2011, a total of 11 patients underwent *transoral* repair as opposed to 70 patients who underwent laparoscopic or open revision of a failed fundoplication. Subjective and objective outcomes were evaluated with the GERD health-related quality of life (GERD-HRQL) questionnaire and the reflux symptom index (RSI) questionnaire and ambulatory pH testing. The competency of the new antireflux barrier was evaluated by endoscopy. Wilcoxon signed-rank test was used to compare pre- and postoperative variables.

**Results:**

All 11 patients evidenced loosening of the Nissen fundoplication without evidence of hiatal failure. Mean age was 57 years, BMI was 25.1 kg/m^2^, and 4 of 11 (36 %) were female. Indications for operation were abnormal pH-metry off PPIs (6), impedance/pH on PPIs (3), esophagitis (1), and evidence of free reflux on barium swallow (1). One patient developed a postoperative bleed requiring transfusion. Two patients had laparoscopic revision at 6 and 8 months after the transoral procedure. At a median follow-up of 14 (range = 6–28) months, 8/10 patients reported resolution of their primary symptoms. Eight patients had pH testing off PPIs both pre- and postoperatively; median % time with pH <4 improved by dropping from 8.1 % (21–4.8 %) to 0.6 % (13.4–0.01 %) (*p* = 0.008). Esophageal acid exposure normalized in 5/6 patients. Mean GERD-HRQL score improved significantly by dropping from 28.6 (10.6) preoperatively to 6.7 (6.1) post-TIF (*p* = 0.016). Mean RSI score improved more than 50 % in 5/7 patients.

**Conclusion:**

Transoral revision of failed traditional fundoplication without herniation is technically feasible. It results in symptomatic and objective improvement of GERD without the risks of laparoscopic dissection for a majority of patients.

Recurrent gastroesophageal reflux after antireflux surgery (ARS) occurs in some patients due to loosening of the fundoplication without anatomic hiatal failure, i.e., transthoracic migration of the wrap. Laparoscopic revision of failed fundoplication is certainly feasible; however, the surgery is difficult and involves risk of gastric, esophageal, and vagal nerve injury that is higher than the risk with primary fundoplication. Systematic reviews of laparoscopic revision of failed ARS report postoperative complication rates up to 44 %, even in highly specialized centers [1, 2]. Data from nonspecialized centers are lacking and fail to support results obtained by specialty centers.

The EsophyX^®^ device (EndoGastric Solutions, Inc., Redmond, WA, USA) offers an alternative, less invasive, transoral approach to revision of loose fundoplication with avoidance of the dissection and risks associated with conventional revision. The purpose of this study was to assess the feasibility and safety of the transoral approach to revision of loose fundoplication. In addition, the study aimed to determine the objective and subjective outcomes after transoral revision. To our knowledge, this is the first series reporting transoral revision of failed traditional antireflux surgery.

## Methods

This report is a retrospective review of prospectively collected and maintained data with institutional review board approval. All patients enrolled in this study signed an informed consent form.

### Patients

Beginning in 2009, a total of 14 patients who had previously undergone a traditional primary or revisional Nissen fundoplication and had symptomatic and objective evidence of recurrent GERD due to loosening of the fundoplication without any evidence of hiatal failure were evaluated for revisional procedure. Eleven of 14 patients elected to have the transoral revision as opposed to laparoscopic/open revision after giving full informed consent.

### Study variables

To determine the feasibility of the transoral approach to revision of loose fundoplication, duration of the transoral incisionless fundoplication (TIF) procedure, valve characteristics (circumference and length), and the number of contributing sutures (fasteners) were recorded. Serious adverse events and the complication rate were used to assess the safety of the procedure. Resolution of primary symptoms, healing of esophagitis, complete elimination of proton pump inhibitor (PPI) use, change in esophageal acid exposure and number of reflux episodes, and normalization or a ≥50 % reduction as indicated in disease-specific validated questionnaires were used to assess the clinical outcomes.

### Preoperative assessment

All patients underwent endoscopy, barium esophagram, or ambulatory pH reflux testing to confirm recurrent reflux. Typical GERD symptoms were assessed with the GERD health-related quality of life (GERD-HRQL) questionnaire and atypical symptoms were assessed with the reflux symptom index (RSI) questionnaire. These two surveys measure GERD symptoms on the visual analog scale from 0 (no symptoms) to 5 (worst symptoms) [3, 4].

### Revisional procedure

All revisional procedures were performed with the EsophyX^®^ device and corresponding TIF 2.0 technique that has been described extensively in a prior publication [3]. In our view, the use of this technology for the revisional procedure is within FDA-cleared indications.

Unlike a primary TIF procedure, revision of a Nissen procedure involves rebuilding a previously constructed artificial valve. Failure of the previous fundoplication involves shortening and loosening of the wrap and can be visualized as having a fundoplication created with only one suture, and that one suture was tied loosely. Reconstruction attempts to restore the valve to its earlier length and to restore the circumference back to over 300°.

Upon introduction of the device, the circumference of the valve is assessed visually. If the circumference is less than 300°, then the helix is engaged near the anterior lip of the valve and placed under caudal traction. The tissue mold is closed to the point that it contacts the cranial limit of the existing fundoplication. The stomach is partially desufflated and the tissue mold and helix are rotated counterclockwise (screen image) toward the lesser curve, with caudal tension maintained on the helix. The tissue mold and helix are then locked down and one set of fasteners is deployed. Generally, this will rotate the anterior lip of the fundoplication more toward the lesser curve and simultaneously lengthen the valve. The maneuver is repeated another one to three times, placing fastener sets at various depths along this newly constructed anterior groove.

A mirror-image procedure is performed on the posterior corner with two to four sets of fasteners.

Having restored as much circumference to the valve as possible, the portion of the valve between the anterior and posterior corners likely will still be effaced. The helix is engaged in this flat lip at two to three sites and, with caudal tension on the helix, the tissue mold is closed and fasteners are deployed as cranially as possible to lengthen the valve.

Tissues are more fibrotic in revisional procedures and less rotation is possible compared to a primary TIF procedure. However, there is more length to work with in a revisional surgery than in a primary TIF, as the prior fundoplication had created length before its loosening and flattening.

### Intraoperative assessment, postoperative care, and follow-up assessment

Intraoperatively, the number of contributing fasteners, their location, and the duration of the procedure were recorded. Intraoperative endoscopy assessed the overall valve characteristics. The length and the circumference of the wrap were recorded.

Discharge date was recorded. Patients were asked to follow standard post-TIF diet (liquid for the first 2 weeks; soft diet the following 2 weeks; slowly transitioning to a normal diet after 4 weeks) and continue PPI medication for at least 2 weeks to assist gastric mucosal healing.

At the follow-up, to evaluate the clinical outcomes patients were asked to complete the GERD-HRQL and RSI questionnaires and undergo ambulatory pH testing. GERD medication use was recorded as “none” or “daily.”

### Statistical analysis

Prospectively collected data were analyzed using JMP statistical software revision 9.0 (SAS Institute Inc., Cary, NC, USA) and presented as medians with ranges and means with standard deviations (SD). Wilcoxon signed-rank test was used to compare pre- and postoperative variables. A *p* value <0.05 was considered significant.

## Results

A total of 11 patients underwent transoral revision of a prior Nissen fundoplication. One patient underwent laparoscopic Nissen fundoplication at another institution 6 months post-transoral revision due to persistent GERD symptoms. This patient did not complete follow-up and was considered a failure. One patient who completed the 6-month follow-up developed a paraesophageal hernia requiring laparoscopic revision 7 months after TIF; pH testing prior to repair of the paraesophageal hernia showed 0.1 % esophageal acid exposure.

### Baseline characteristics

Of the 10 patients who completed follow-up, 64 % were male. Median body mass index (BMI) was 24.4 (range = 15.9–42.0) kg/m^2^. All patients had objective evidence of GERD by esophagitis (1), free reflux on barium esophagram (1), pH testing off PPIs (6), or impedance/pH testing on PPIs (3). One patient was operated on solely on the basis of free reflux on barium esophagram. In our experience, free reflux is very unusual in a patient with an intact fundoplication. Presenting primary typical symptoms were heartburn in 7 and regurgitation in 3 patients at a median of 2.8 (range = 1–16) years after primary (7) or revisional (3) Nissen fundoplication. Secondary symptoms were dysphagia (1), aspiration (2), asthma (2), dyspnea (1), sore throat (1), hoarseness (1), throat clearing (1), and cough (1). Esophagitis was present in one patient, five patients had a hiatal hernia ≤2 cm, and no patients had Barrett’s esophagus. All patients were on daily PPIs. Baseline characteristics of the study population are presented in Table 1.

**Table 1:** Baseline characteristics of study population (*n* = 10)

Data	Age	BMI	Years since prior Nissen	PPI daily dose before TIF	Regurgitation score	Total GERD-HRQL score	Reflux symptom index score
Median	60	24.4	2.8	60.0	12.0	28.5	19.0
Max	75	42.0	15.6	120.0	27.0	45.0	37.0
Min	28	15.9	0.8	20.0	0.0	16.0	6.0
Mean	56.6	25.1	5.3	60.0	13.4	31.1	21.9
SD	13.5	6.4	5.2	28.3	9.4	14.7	10.2

### Operative outcomes

A total of 11 TIF procedures were completed. At the beginning of the procedure, prior to introduction of the EsophyX^®^ device, flexible esophagogastroscopy was performed to carefully look for evidence of a paraesophageal hernia or of an enlarged diaphragmatic impression in retroflex view. One patient very desirous of TIF was found to have a slightly enlarged hiatal impression of 3 cm and simultaneously underwent anterior closure of the slightly enlarged hiatus (one suture). No other patients underwent laparoscopic evaluation. One patient stayed >24 h in the hospital due to postoperative hypoxemia. One patient, early in our experience, developed intraoperative intraluminal bleeding from the site of dislodgement of the helical retractor and required transfusion. Intraoperative variables of interest are presented in Table 2.

**Table 2:** Intraoperative variables of interest

Data	Time	Intact fasteners	Anterior	Midanterior	Posterior	Final degree wrap	Final valve length
Mean	64.50	19.2	6	6	10	311	3.7
Max	90.00	25	6	8	10	340	5.0
Min	41.00	11	6	4	9	300	3.0
Median	63.50	21	6	6	10	300	3.5
SD	18.66	4.6	0	3	1	15	0.6

### Subjective outcomes

At a median follow-up of 14 (range = 6–28) months, eight of ten patients reported resolution of their primary (typical) GERD symptoms and nine were off acid-suppressive therapy. Only one patient remained on PPI therapy after transoral revision of Nissen fundoplication despite a pH test demonstrating 0.5 % esophageal acid exposure. Seven patients had completed the GERD-HRQL and RSI questionnaires before and at a follow-up visit. Total GERD-HRQL scores (calculated per Velanovich) and total RSI scores normalized or improved by ≥50 % in five of seven patients (Table 3). No de novo dysphagia, bloating or flatulence was reported.

**Table 3:** GERD health-related quality of life (GERD-HRQL), reflux symptom index (RSI), heartburn, regurgitation, bloating, and flatulence scores before transoral incisionless fundoplication and at the median of 14 (6–28) months after surgery

Scores	Preoperative mean	Postoperative mean	*p* Value*
GERD-HRQL total	28.6	6.7	0.016
GERD-HRQL heartburn	17.9	5.3	0.016
Regurgitation	11	3	0.125
RSI	20.6	9.1	0.031
Bloating	2.4	0.7	0.031
Flatulence	2.9	1.4	0.031

Values are mean (standard deviation)

* *p* Values are calculated using Wilcoxon signed-rank test

### Objective outcomes

**Fig. 1:**
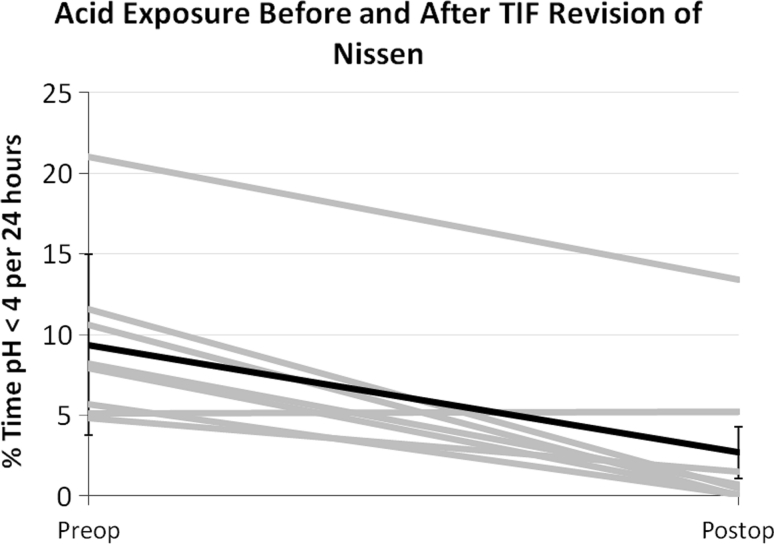
Esophageal acid exposure before and after TIF revision of Nissen. *Gray lines* represent individual patients, *black line* represents mean with standard deviation. Testing performed with the patient off acid suppressive medication

**Fig. 2:**
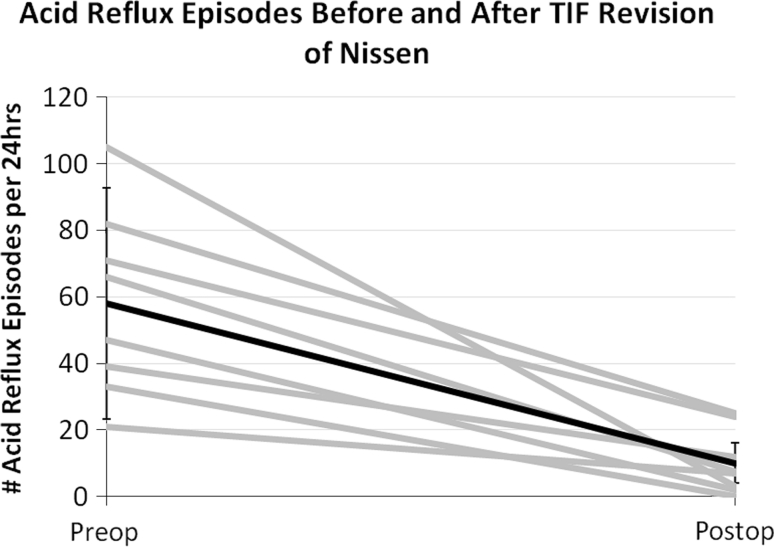
Number of acid reflux episodes per 24 h before and after TIF revision of Nissen. *Gray lines* are individual patients, *black line* represents mean with standard deviation. Testing performed with patient off acid suppressive medication

Eight patients had pH testing while off acid-suppressive medication pre- and postoperatively. In these eight patients, the median % time with pH <4 improved by dropping from 8.1 % (4.8–21.0 %) to 0.6 % (0.0–13.4 %) (*p* = 0.008, Wilcoxon signed-rank test) (Fig. 1). The median number of reflux episodes per 24 h decreased from 66 (range = 21–105) to 23 (7–26) (*p* = 0.03) (Fig. 2). Of the eight patients with pH testing while off PPIs, six (75 %) presented with abnormal % time with pH <4 preoperatively (>5.3 %); five of six (83 %) had normalized.

## Discussion

A mechanical cause for anatomic failure of a fundoplication could be transthoracic migration of the fundoplication, loosening of the fundoplication, or both. In one review, 23 % of 3,175 failures were due to isolated loosening of the fundoplication [2] and in another review it was 18 % [1]. This is in keeping with our experience that roughly 20 % of failures have been due to simple loosening or flattening of the fundoplication without any evidence of paraesophageal herniation of the wrap. In some cases a small axial hernia component may exist; however, transoral revision of these failures does not require closure of the hiatus. If roughly 20 % of primary operations fail and 20 % of these are due to isolated loosening of the fundoplication, then 4 % of primary Nissen would eventually be candidates for TIF revision.

Revisional surgery of a loose fundoplication is not an easy task. It is difficult, tedious, and places the esophagus, stomach, and vagus nerves at increased risk due to the adhesiolysis. Systematic reviews found that intraoperative esophageal or gastric injury occurred in 13 % of 2,123 open or laparoscopic reoperations [2], and there was an overall perioperative complication rate of 14 % (range = 0–44 %) of 810 laparoscopic reoperations [1]. Hospital stays are longer after laparoscopic redo surgery than after primary surgery [1]. Symptomatic success rates average from 81 % [2] to 84 % [1]. Objective outcomes were measured in only 13 % of patients, with a “successful” outcome in 78 % [2], with only four laparoscopic studies reporting clearly defined objective outcomes [4–7]. The authors of one review concluded that the relatively disappointing results of redo antireflux surgery support the opinion that redo surgery is tertiary referral center surgery [2].

We started performing TIF at the beginning of 2009. With increased experience in both device manipulation and transoral fundoplication, we thought that the transoral revision of a loose Nissen fundoplication may benefit patients with recurrent GERD symptoms who were unwilling to undergo another laparoscopic procedure. We hypothesized that the transoral approach, used primarily to create a de novo fundoplication, might be effective in restoring a loose Nissen fundoplication. This report of 11 patients who underwent a TIF revision of a prior Nissen procedure is, to our knowledge, the first consecutive series to be published.

**Fig. 3:**
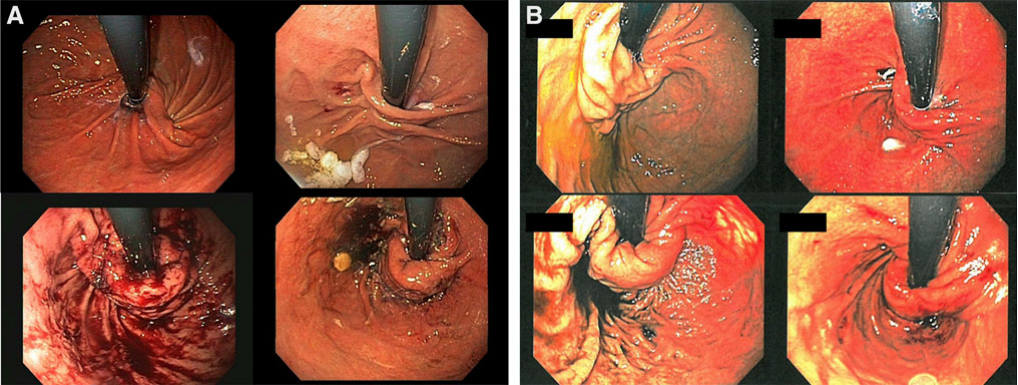
Endoscopic view of the gastroesophageal junction before (*top*) and after revision (*bottom*). **A** TIF revision. **B** Laparoscopic Nissen revision

Our intention was to evaluate the feasibility and safety profile of the transoral repair of a loose Nissen fundoplication. The transoral revision technique was straightforward and probably easier than a primary TIF. Visually, recurrent reflux, especially in the absence of transthoracic wrap migration, appears as flattening and loosening of the fundoplication (Fig. 3). If such a patient were to undergo laparoscopic revision, intraoperative endoscopy would demonstrate restoration of length and circumference (personal observation). The characteristics (length and circumference) of the TIF revisional wrap approached those seen with a laparoscopically revised fundoplication (Fig. 3). The goal of this study was not to investigate the associations between the valve vectors (circumference, length, and the number of fasteners used) and the clinical outcomes. Anecdotal evidence suggests that a valve ≥3 cm long and with a circumference ≥270° will render acceptable symptomatic relief. If anatomic condition permits, we tend to use a greater number of fasteners (~20) to reconstruct a more robust and tight valve. The median time necessary to perform the revision (63 min) was in line with the time required to perform a de novo transoral fundoplication and significantly less than the average time for laparoscopic reoperation (164 min) [1].

One patient in this series, early in our overall TIF experience, had a postoperative bleed that required transfusion, for a complication rate of 9 %. This was due to traumatic dislodgement of the helical retractor during device manipulation. The site of the hemorrhage was along the greater curve aspect of the fundoplication at the lip of the valve. From this event we learned three things: (1) minimize the number of helical retractor engagements and be mindful of its potential to be dislodged during repositioning; (2) have endoluminal hemostatic devices available and to be knowledgeable in their use; (3) that the increased fibrosis in revisional procedures may increase the risk of postoperative hemorrhage. Further experience with the device and attentiveness to these three things has resulted in no bleeding in more than 100 patients who underwent TIF afterward. This complication appears avoidable with knowledgeable use of the device. The other minor complications, consistent with a primary TIF procedure, were left shoulder pain, sore throat, nausea, and abdominal and epigastric pain. However, these minor complications were resolved shortly after the surgery. Only one patient stayed in the hospital for >1 day due to postoperative hypoxemia.

The clinical results in these 11 patients have been very encouraging, and on par with what are seen with laparoscopic revision [8], with relief of primary symptoms and cessation of PPI use in over 70 % of patients at 14 months follow-up. Quality-of-life assessments confirm significant reduction in heartburn, regurgitation, and laryngopharyngeal reflux symptoms and absence of de novo dysphagia, gas bloat, and flatulence. Additionally, objective measures of the results of transoral revisions are in line with what is seen after laparoscopic fundoplication and attest to the restoration of the Nissen valve in 9/11 (81 %) of our patients, based on endoscopic evaluation [9, 10].

Limitations of this study are its retrospective nature and the limited number of patients (although 5 of the 20 available studies in a recently published systematic review had 10 or fewer patients) [1]. The median follow-up of 14 months is on par with many of the published reports of laparoscopic redo antireflux surgery, but on the lower end. Seventy-three percent of patients underwent pre- and postoperative reflux testing, which is a greater percentage than in most other studies of laparoscopic revision. Additionally, this was a single-center, single-surgeon study and the results might not be representative of the large population. Specifically, the relatively high percentage of patients with normalized pH postoperatively (83 %) is based on only six patients with abnormal esophageal acid exposure before revision. A large prospective randomized trial comparing the laparoscopic and transoral approaches would be ideal but may be difficult to conduct due to difficulties in standardizing both treatment approaches and the inability of the device to reduce hiatal hernia >2 cm. However, carefully designed single- and multicenter single-arm studies to evaluate the safety and efficacy of transoral repair of failed Nissen fundoplication are desirable. Based on our results, the transoral repair of a failed traditional fundoplication is worth considering at the centers that invest the time necessary to master the transoral procedure.

## Conclusions

Although based on a small population, this first published series of a transoral approach to revise loose Nissen fundoplications demonstrated its safety and had subjective and objective results on par with laparoscopic revision of failed Nissen fundoplications. With proper attention to technique, we believe the safety of a transoral revisional fundoplication may be greater than that of a laparoscopic revisional fundoplication.

## Abbreviations

GER: Gastroesophageal reflux
GERD: Gastroesophageal reflux disease
GERD-HRQL: Gastroesophageal reflux disease health-related quality of life
RSI: Reflux symptom index
TIF: Transoral incisionless fundoplication
